# Basonuclin-Null Mutation Impairs Homeostasis and Wound Repair in Mouse Corneal Epithelium

**DOI:** 10.1371/journal.pone.0001087

**Published:** 2007-10-31

**Authors:** Xiaohong Zhang, Hung Tseng

**Affiliations:** 1 Department of Dermatology, University of Pennsylvania, Philadelphia, Pennsylvania, United States of America; 2 Cell and Developmental Biology, University of Pennsylvania, Philadelphia, Pennsylvania, United States of America; 3 Center for Research on Reproduction and Women's Health, University of Pennsylvania, Philadelphia, Pennsylvania, United States of America; University of Hong Kong, China

## Abstract

At least two cellular processes are required for corneal epithelium homeostasis and wound repair: cell proliferation and cell-cell adhesion. These processes are delicately balanced to ensure the maintenance of normal epithelial function. During wound healing, these processes must be reprogrammed in coordination to achieve a rapid re-epithelialization. Basonuclin (Bnc1) is a cell-type-specific transcription factor expressed mainly in the proliferative keratinocytes of stratified epithelium (e.g., corneal epithelium, epidermis and esophageal epithelium) and the gametogenic cells in testis and ovary. Our previous work suggested that basonuclin could regulate transcription of ribosomal RNA genes (rDNA) and genes involved in chromatin structure, transcription regulation, cell-cell junction/communication, ion-channels and intracelllular transportation. However, basonuclin's role in keratinocytes has not been demonstrated in vivo. Here we show that basonuclin-null mutation disrupts corneal epithelium homeostasis and delays wound healing by impairing cell proliferation. In basonuclin-null cornea epithelium, RNA polymerase I (Pol I) transcription is perturbed. This perturbation is unique because it affects transcripts from a subset of rDNA. Basonuclin-null mutation also perturbs RNA polymerase II (Pol II) transcripts from genes encoding chromatin structure proteins histone 3 and HMG2, transcription factor Gli2, gap-junction protein connexin 43 and adheren E-cadherin. In most cases, a concerted change in mRNA and protein level is observed. However, for E-cadherin, despite a notable increase in its mRNA level, its protein level was reduced. In conclusion, our study establishes basonuclin as a regulator of corneal epithelium homeostasis and maintenance. Basonuclin likely coordinates functions of a subset of ribosomal RNA genes (rDNA) and a group of protein coding genes in cellular processes critical for the regulation of cell proliferation.

## Introduction

The adult mouse cornea is comprised of three tissue layers: a surface of stratified epithelium, a collagenous stroma underneath and an inner endothelium. The corneal epithelium is a self-renewing tissue, whose maintenance requires continuous cell proliferation throughout life. In addition to its light refraction function, corneal epithelium also serves as a protective barrier to the external environment. This barrier function makes corneal epithelium susceptible to a variety of mechanical and chemical injuries. Conceivably, a corneal epithelium that can rapidly repair wounds would convey selective advantages to animals, whose survival relies on vision.

Two cellular processes are essential for corneal epithelium maintenance and wound repair: cell proliferation and cell adhesion. In normal renewing, corneal epithelium stratification (thickness) is maintained by a balance between cell proliferation at the basal layer and cell shedding at the surface. This life-long continuous upward cell flux is driven by the corneal epithelium stem cells resided in the limbus region [1]–[4]. Accompanying the surface-ward cell migration is a differentiation process, which involves, among other things, remodeling of cell-cell junction and communication (e.g., formation of tight junctions, gap junctions and ion channels in the suprabasal wing cells) [5]–[9]. In wound healing, however, these processes are modified in order to achieve a rapid re-epithelialization [10]. In the commonly used wound healing model (i.e., epithelial debridement at central cornea), the healing process is divided into three sequential and partially overlapping steps: cell migration reduces and eventually covers the wound surface, cell proliferation provides cells to rebuild the tissue and tissue remodeling to restore the stratified epithelium [9], [11]–[16].

Basonuclin (Bnc1) is a zinc finger protein with highly restricted tissue/cell distribution [17], [18]. It is present in abundance in only two types of cells: basal keratinocytes of stratified epithelium (e.g., corneal epithelium and epidermis), and the reproductive germ cells of testis and ovary (i.e., oocytes and spermatogenic cells) [19], [20]. In mouse corneal epithelium, basonuclin expression is restricted to the basal keratinocytes in the central cornea; it is not detected in the limbal region, suggesting its absence in the multi-potent stem cells [21]. Basonuclin protein appears in the corneal basal keratinocyte at post-natal day 4, first in a few cells and then spread to the entire basal cell population on day 20 [21]. Thus, in early neonatal period, basonuclin expression coincides with active cellular proliferation, which peaks at day 10 prior to eye opening [15]. This expression pattern and the basal-cell location of basonuclin strongly suggested its role in cell proliferation [18], [21].

Basonuclin is a transcription factor with multiple functions. Biochemically, DNase I footprinting showed that basonuclin interacted with ribosomal RNA gene (rDNA) promoter at three highly conserved binding sites in human and mouse [22]–[25]. Consistent with this interaction, basonuclin remained at the rDNA loci during mitosis [23], a typical behavior of transcription factors for Pol I, which is solely responsible for transcribing rDNA. Furthermore, basonuclin zinc fingers were shown to act as a dominant-negative agent to inhibit Pol I transcription in mouse oocytes [24]. These observations strongly suggest that basonuclin plays a role in Pol I transcription regulation. Basonuclin is unique among the known regulators of Pol I transcription in that its expression is cell-type-specific and it functions in regulating a subset of rDNA. Such a novel ability was first hinted in our study of basonuclin-deficient oocytes. Run-on assays showed that in basonuclin-deficient oocytes, the number of Pol I transcription foci was reduced by ∼25%, and the transcriptional activity of the remaining foci, as judged by BrU incorporation, was not changed [26]. Similarly, we showed in HaCaT cells, a transformed human keratinocyte line, that basonuclin interacted with rDNA promoters that differed from those bound with UBF, a ubiquitous Pol I transcription regulator [25]. These observations support the notion that basonuclin regulates a subset of rDNA and lead us to search for rDNA variants that are regulated cell-type-specifically. As a result, we isolated seven rDNA variants (v-rDNAs) and showed, for the first time, that they were regulated differentially in different tissues (Tseng et al. submitted).

Another major difference of basonuclin and a dedicated Pol I transcription factor (e.g., UBF) is that basonuclin is also abundant in nucleoplasm and can interact with Pol II promoters. Indeed, a recent bioinformatic study detected basonuclin binding sites in the promoter regions of more than 20 genes, which belong to pathways involved in chromatin structure, transcription, cell-cell adhesion/communication, ion-channels and intracellular protein transportation [27]. The dual function of basonuclin to regulate two groups of genes, (i.e., Pol I- and Pol II-transcribed) suggests that it coordinates the function of these genes. Thus, basonuclin is likely a coactivator-like regulator. The concept of coactivator is that a regulator can mobilize multiple pathways to achieve a cellular function [28]. The coactivators were initially thought to function as adaptors for transcription factors [29], but their actions have now expanded to chromatin modification and remodeling, transcription initiation, elongation, RNA splicing and protein degradation. The range of target pathways of coactivators has also increased to encompass cell development, proliferation, energy-expenditure and carbohydrate- metabolism [30]–[32]. These coactivators are therefore coordinators of multiple cellular pathways.

Despite these advances in understanding basonuclin function, its requirement in keratinocyte function has not been studied in vivo. Here we report the construction of a basonuclin-null mouse and characterization of basonuclin-null corneal epithelium. We show that basonuclin-null mutation impairs keratinocyte proliferation. We present in vivo evidence supporting the notion that basonuclin regulates transcription of a subset of rDNA and a selective group of protein-coding genes. We propose that basonuclin is a critical regulator for corneal epithelium homeostasis.

## Results

### Construction of Bnc1-null mouse

**Figure 1:**
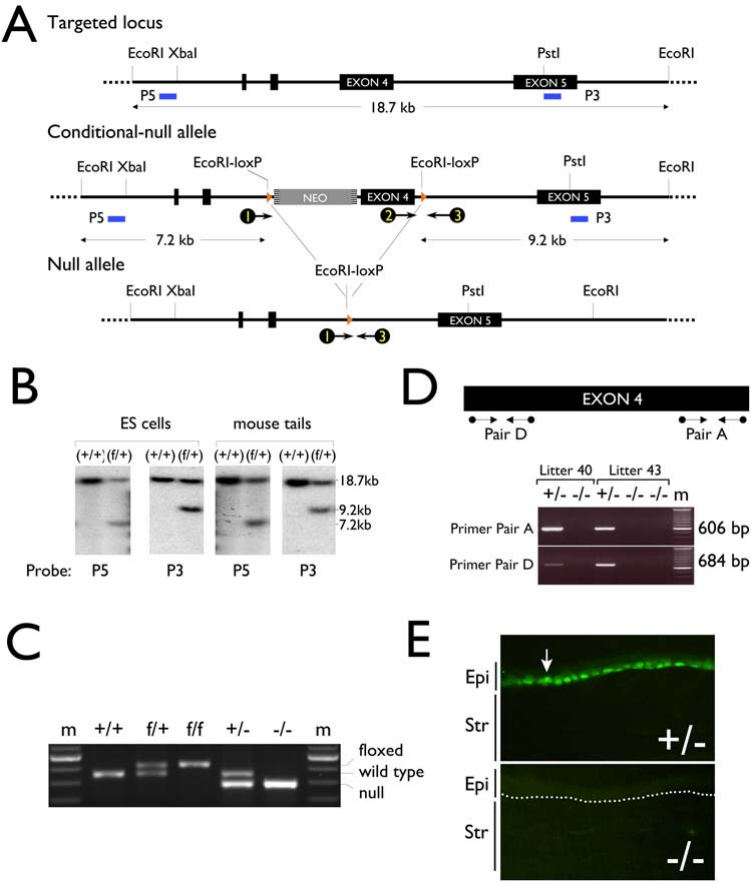
Generation of basonuclin-null mouse. A, A graphic depiction of the knockout strategy. The targeted locus (∼18.7 kb) of the basonuclin gene is shown at the top. Restriction sites relevant to cloning and detection of the conditional allele are indicated. Exons are depicted as black boxes and the Southern probes (P5 and P3) are indicated as short blue lines. Shown below the targeted locus is the structure of basonuclin conditional-null allele, which consists of a *neo* gene upstream from the Exon 4. The *neo* gene and the Exon 4 are flanked by loxP sites. To facilitate the detection of the conditional-null allele, two new Eco RI sites are generated with the insertion of the lox P sites. Upon Eco RI digestion, the wild type allele produces a single 18.7 kb fragment, whereas that of the conditional-null allele, two fragments of sizes 7.2 kb (detected by 5′ probe) and 9.2 kb (3′ probe). The null-allele, which is generated after the excision of the *neo* and the Exon 4 by Cre recombinase, is shown below the conditional-null allele. PCR primers, depicted as circle-ended arrows, are designed to facilitate genotyping. B, Successful homologous recombination of the targeting vector was verified by Southern blots. Genomic DNAs from ES cells and founder mice tails were digested with Eco RI and analyzed by Southern blot using 5′ (P5) and 3′ (P3) probes (A, blue lines) outside the targeted region (defined by the lox P sites). The genotypes are indicated at the top of the Southern image, molecular weight markers are shown to the right and the probes at the bottom. f, the allele with inserted loxP. C, The null allele was generated by mating the female conditional knockout mice with ZP3-Cre males to create a Bnc1 (flox/+);ZP3-Cre female, which was then crossed with normal males. The genotypes of Bnc1 (+/+) (f/+), (f/f), (+/−), and (−/−) mice were identified by PCR and the deletion of Exon 4 was verified by PCR with primers indicated in A. In the PCR, all three primers were present, primers 2 and 3 detected the wild type and conditional alleles and primers 1 and 3 detected the null allele. The primer extension step was limited so that primers 1 and 3 could not produce a fragment with the wild type and the conditional allele. floxed, loxP-inserted. D, The absence of basonuclin mRNA was demonstrated with two pairs (A and D) of PCR primers targeting different regions of the Exon 4 (top panel). Tail RNAs of three Bnc1 (−/−) mice from two litters were assayed. The litters and genotypes are indicated at the top of the gel image and molecular weight of the amplicon on the right. E, The absence of basonuclin protein was demonstrated by immunostaining. The arrow shows an example of a basonuclin-positive cell. A dotted line indicates the position of basement membrane. Epi, epithelium; Str, stroma.

We designed a basonuclin conditional-null allele, in which Exon 4 was targeted for deletion in the presence of Cre recombinase (**Fig. 1**). The conditional-null allele consisted of Exon 4 and a *neo* gene, which served as a selective marker (**Fig. 1A**). The Exon 4 and the *neo* were flanked by LoxP sites, where Cre recombinase-mediated deletion occurred. The deletion created a stop-codon in the remaining reading frame to prevent translation of sequence beyond the deletion site. Thus, the null-mutation removed approximately 90% of basonuclin protein sequence, which included all six zinc fingers and the nuclear localization signal. Such deletion would prevent the remaining 10% sequence from entering the nucleus [33].

The targeting construct was transduced into ES cells (R1 strain, [34]) via electroporation. The transduced cells were subjected to G418 (*neo*) selection. In approximately 3% of the G418-resistant ES-cell clones, the conditional-null allele (fl-neo) replaced the normal allele. This conclusion was based on observing the predicted 7.2 kb and 9.2 kb Eco RI fragments by Southern analysis using a 5′ (P5) and a 3′ (P3) probes, respectively (**Fig. 1A, B**). Germ line transmission of the conditional-null allele was shown by Southern blots (**Fig. 1B**) and by PCR (**Fig. 1C**). Bnc1 (+/−) mice were obtained by mating the Bnc1 (+/fl-neo) with a ZP3-Cre mouse, which expressed Cre recombinase only in the oocytes [35]. As expected, approximately 50% progeny were Bnc1 (+/−), which were apparently normal and fertile. The Bnc1 (+/−) mice were crossed to obtain basonuclin-null mice (Bnc1 −/−). PCR detected Bnc1 −/− progeny (**Fig. 1C**), which were born at a reduced frequency (not shown) and the surviving pups developed normally except infertile. (The infertility phenotype will be described elsewhere.) In the total RNA of basonuclin-null tail, RT-PCR detected no mRNA sequence from Exon 4 (**Fig. 1D**). Furthermore, immunocytochemistry confirmed the absence of basonuclin protein in the basal keratinocytes of corneal epithelium, where it was normally expressed (**Fig 1E**). We thus successfully ablated the basonuclin gene. Because this whole-body basonuclin-knockout mouse was viable, we used them for the experiments described here and did not generate a corneal-specific basonuclin-null mutant.

### Basonuclin-null corneal epithelium is thinner

**Figure 2:**
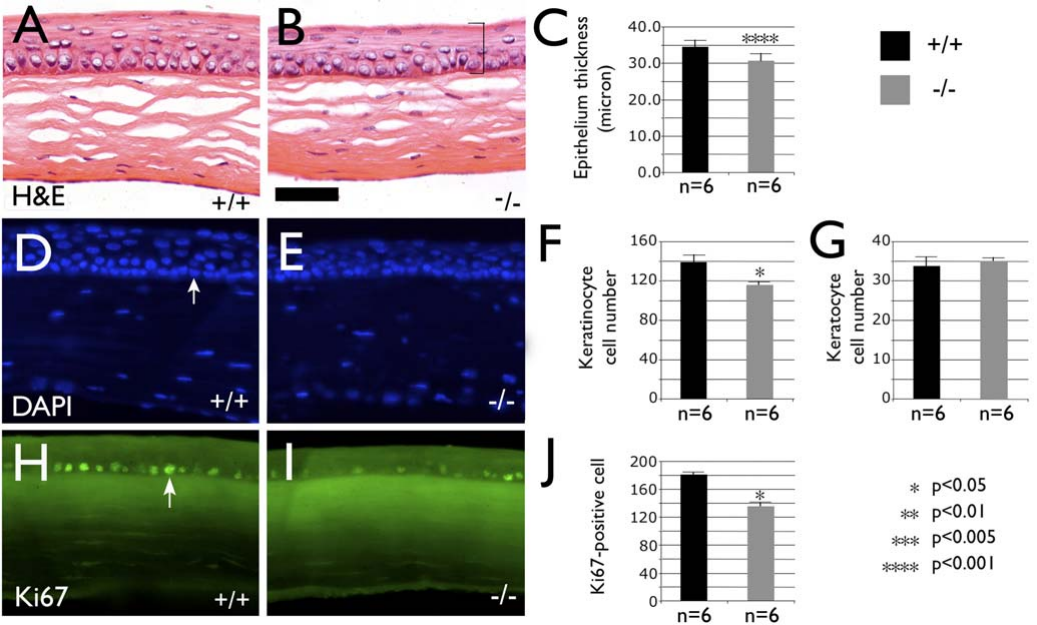
Basonuclin-null corneal epithelium is thinner and contains fewer cells. Compared with the histology (H & E) of normal cornea (A), basonuclin-null cornea (B) is thinner (a bracket shows the definition of epithelial thickness. C, Measurements of the corneal epithelium thickness show the thinning caused by basonuclin-null mutation is highly significant (n = 6, p<0.001). DAPI staining of nuclear DNA (the arrow shows an example) shows that compared with the control (D), basonuclin-null (E) corneal epithelium has ∼15% fewer cells (F) (n = 6, p<0.05), whereas the cell numbers in stroma are the same (G). The reduced epithelial cell number is likely caused by a reduced cell proliferation. Compared with the control (H), the basonuclin-null (I) epithelium contained ∼25% fewer Ki67 antigen-positive cells (J) (n = 6, p<0.05). The arrow in H shows an example of Ki67-positive cells. The statistical analyses represent the average of six mice for each genotype. The code of statistical significance is given at the lower right corner. All images are of the same magnification. Scale bar in B equals 30 micron.

Corneal epithelium was present in basonuclin-null mice, suggesting basonuclin was not critically required for its development. We therefore examined corneal epithelium homeostasis in 12-week old mice, when corneal epithelial development had largely completed [36]. Histology (hematoxylin and eosin, H&E) showed a 14% thinning of basonuclin-null corneal epithelium, which was on average 30.6 microns thick compared with the control's 34.6 microns (for each genotype n = 6, p<0.001)(**Fig. 2A, B, C**). By counting 4,6-diamino-2-phenylindole (DAPI)-stained nuclei, we found that the thinner epithelium contained 15% fewer cells compared with the control (i.e., Bnc1 +/+). The average cell density was 116±8 (cell number per 0.3 mm corneal epithelium in length) in basonuclin-null corneal epithelium compared to that of 139±11 in the control (n = 6, p<0.05) (**Fig. 2D, E, F**). In contrast, no difference in cell density was seen in corneal stroma of the mutant and the control (n = 6)(**Fig. 2G**).

The reduced cell number could be the result of a decrease in cell proliferation or an increase in apoptosis. To discern these possibilities, we examined the number of Ki67-positive cells by immunostaining. Ki67 antigen was a marker for cell proliferation; it was expressed in every stage of the cell cycle, but absent in cells that had exited permanently from the cell cycle. The number of Ki67-positive cells was scored across the entire section of corneal epithelium. Corneas from six mice of each genotype (Bnc1 +/+ and −/−) were examined. The average number of Ki67-positive cells was 135±25 per section in basonuclin-null epithelium, compared with 181±17 in the control, (n = 6, p<0.05) (**Fig. 2H, I, J**), which translated into a 25% decrease in the mutant. TUNEL assay was used to assess apoptosis. A very small number (<3) of TUNEL-positive cells were detected per corneal section in both basonuclin-null and control epithelium (not shown), indicating that loss of basonuclin did not lead to a significant change in the rate of apoptosis. Therefore, the thinning of basonuclin-null corneal epithelium was likely caused by a reduced cell proliferation.

### Basonuclin-null mutation delays corneal epithelial wound healing

**Figure 3:**
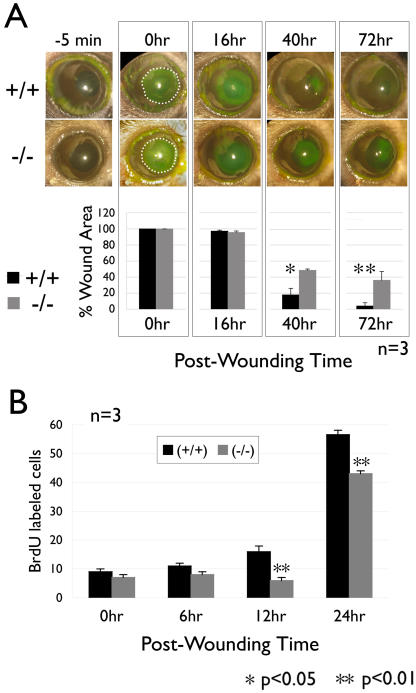
Wound healing is delayed in basonuclin-null corneal epithelium. A, the upper panel shows representative images of the control (+/+) and basonuclin-null (−/−) eyes at different time points pre- and post-wounding. The images of –5 min show the eyes prior to wounding. The denuded surface of corneal regions was topically stained with fluorescein (green). In the 0 hr images, the denuded area is marked with a dashed line to illustrate the size of the original wound. The lower panel shows the quantification (n = 3) of the wounded area (presented as percentage of the original wound area). Note that the slower healing of basonuclin-null epithelium is observed at 40 hr and 72 hr post wounding, but not before. B, BrdU labeling showed a delayed onset of DNA replication in basonuclin-null corneal epithelium during wound healing. The bar graph represents the average number of BrdU labeled cells per section. Two sections from each of three mice were examined (n = 3). * p<0.05, ** p<0.01.

To investigate if reduced proliferation during normal epithelial homeostasis also affected wound repair, we used a corneal epithelium debriment model to compare the re-epithelialization rate between basonuclin-null mice and the control in vivo. The debriment wound removed the epithelium without damaging the underlining basement membrane. Such an epithelial wound was generally repaired (re-epithelialized) within 72 hours in normal mice [11], [37]. The wounded (denuded) area was visualized by fluorescein staining (**Fig. 3A**) and measured at regular interval to assess the healing progress. In the first 24 hours post wounding, the denuded area was reduced at similar rate between basonuclin-null and the control corneal epithelia. A significant delay in re-epithelialization, however, was observed in basonuclin-null epithelium at 40 and 72 hours post wounding (**Fig. 3A**). At 40 hours, the wound area in basonuclin-null cornea was three times that of the control (three mice per genotype, or n = 3, p<0.05), and at 72 hours, nearly six times (n = 3, p<0.01) (**Fig. 3A**), indicating a slower healing in the mutant at the later stage of re-epithelialization. Epithelium re-epithelialization was usually completed in two phases: first, cell migration reduced the wounded area and then cell proliferation regenerated the epithelium. The significant delay in the latter stage of wound healing suggested a defective cell proliferation. We assessed cell proliferation during wound healing by BrdU labeling, which detected cells in the S phase of the cell cycle. During the first six hours after wounding, the numbers of BrdU labeled cells in the control and basonuclin-null mice were similar to their pre-wounding value (0 hours), indicating cell proliferation had not been activated (**Fig. 3B**). At 12 hours post-wounding, the number of BrdU-positive cells began to increase in the control, but not in basonuclin-null epithelium (n = 3, p<0.01). A significant increase in BrdU incorporation was seen in basonuclin-null cells at 24 hours. The number of BrdU-positive cells was, however, still below that in the control (n = 3, p<0.01). These data suggested that there was a delay in the onset of cell proliferation in basonuclin-null epithelium, which was consistent with the delay in the later stage of re-epithelialization in basonuclin-null cornea. Thus, basonuclin-null mutation impaired cell proliferation of corneal keratinocytes, which could account for both the thinning as well as the delayed wound healing of corneal epithelium.

### Basonuclin-null mutation reduced transcripts from a subset of rDNA

**Figure 4:**
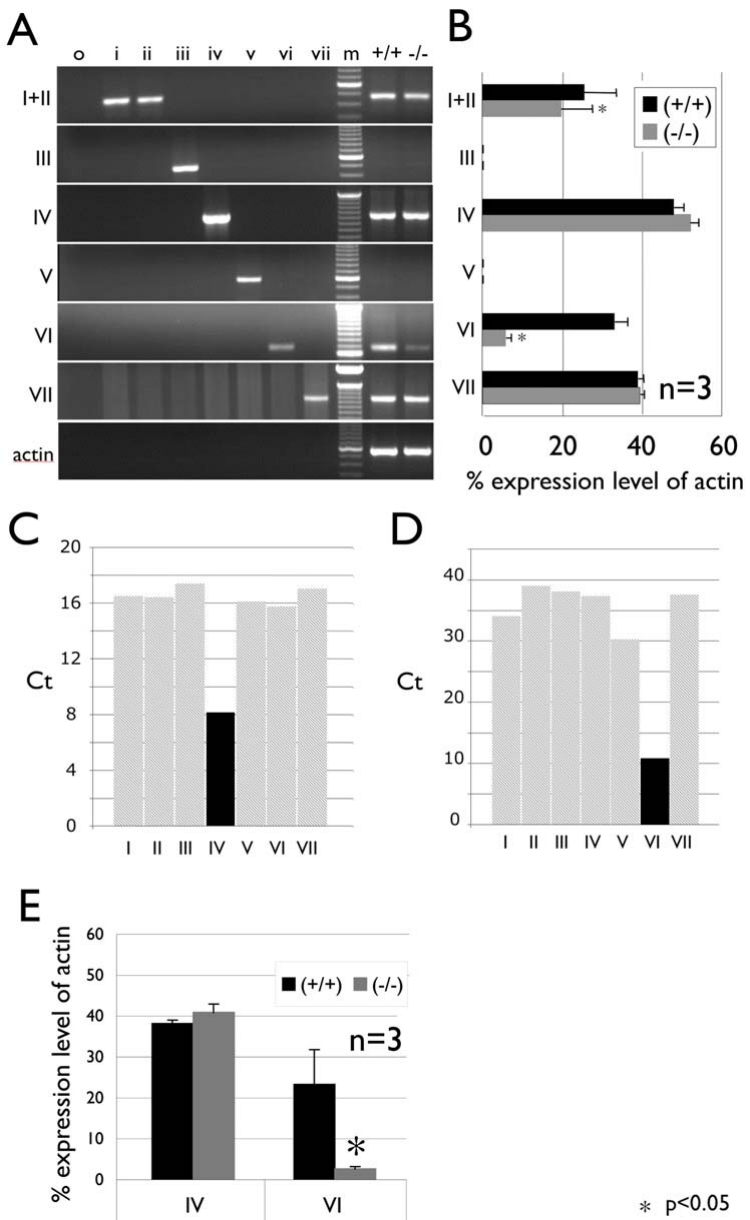
Variant-rDNA expression in basonuclin-null corneal epithelium. A, A set of variant-specific RT-PCRs were used to measure the expression of seven v-rDNAs in the control and basonuclin-null corneal epithelia. The primer specificity (I to VII) is indicated on the left of the gel image; beta-actin PCR was included to monitor the relative quantity and integrity of RNA preparation. The templates are indicated above the gel image: i to vii, cloned v-rDNA controls; m, molecular weight marker (100 bp ladder), RNAs from control (+/+) and basonuclin-null (−/−) epithelia. B, The v-rDNA expression was quantified by measuring the fluorescence of stained PCR product and analyzed statistically. The v-rDNA expression level is expressed as percentage of that of the beta-actin PCR product (100%) (bar graphs). Each value was the average of 3 measurements (n = 3) of a mixture of RNA from four corneal epithelia of each genotype. The results of regular PCR were confirmed by quantitative PCR (real-time PCR or qPCR). C and D, Control experiments were performed to ensure the specificity of qPCR for v-rDNA IV (C) and VI (D). The Ct values of cloned templates are plotted; light gray, non-specific template and black, specific template. A lower Ct value signifies a higher specificity. E, RNA samples similar to that shown in A are measured by qPCR. The v-rDNA expression values are displayed as percentages of the reference gene (beta-actin). * p<0.05, ** p<0.01.

The molecular cloning of seven v-rDNAs (Tseng et al., 2007, submitted) and the construction of the basonuclin-null mouse model provided us with the first opportunity to test the notion that a transcription factor could regulate a subset of rDNA. We had developed a set of PCR assays to detect the expression of each v-rDNA. This assay was based on variant-specific single nucleotide polymorphisms (SNP) located in the 5′ external-transcribed-spacer (5′-ETS) of the 47S pre-ribosomal RNA (pre-rRNA). Using the variant-specific PCR assay, the expression profiles of the seven v-rDNAs were examined in corneal epithelium of basonuclin-null and control mice (**Fig. 4**). For each genotype, total RNAs from four corneal epithelium sheets were pooled and each assay was performed three times (n = 3). Our results showed that corneal epithelium expressed five v-rDNAs (I, II, IV, VI and VII) (**Fig. 4A, B**). This was the first detection of v-rDNA VII transcript, which had not been detected in RNA from all major mouse tissues, and hence v-rDNA VII was previously classified as transcriptionally silent. Basonuclin-null mutation affected the expression of three of the seven v-rDNAs. The expression of v-rDNA I, II and VI was reduced in basonuclin-null mice compared with the control (i.e., Bnc1 +/+). Quantification showed that v-rDNAs I and II transcripts, which could not be distinguished by the assay, were reduced by ∼25% (n = 3, p<0.05) and that of VI more than 80% (n = 3, p<0.05)(**Fig. 4A, B**). The result of v-rDNA VI was confirmed by a real-time PCR assay (**Fig. 4** **, C, D, E**). For a technical reason (insufficient specificity), the result of v-rDNA I+II was not repeated with real-time PCR (not shown). These results were consistent with our previous reports that basonuclin-deficiency affected a subset of rDNA expression [**25**], [**26**]. Our results also identified, for the first time, the rDNA variants that were affected by basonuclin-null mutation.

### Basonuclin-null mutation perturbed the expression of a number of protein-coding genes

The basonuclin target genes transcribed by RNA Pol II were clustered in at least four pathways, i.e., chromatin structure, transcription regulation, ion channels, and cell-cell adhesion [26], [27]. Among these four pathways, we selected 11 target genes for examination by real-time RT-PCR. Each basonuclin target pathway was represented by at least two genes. The selection was also based on target genes' perturbation in basonuclin-deficient oocytes [26], basonuclin's occupancy of the target promoter [27] and the availability and quality of antibodies against the protein encoded by the target gene. To ensure that we detected only mRNA sequences, the primers were chosen to locate in two different exons. Because basonuclin's role in regulating rDNA transcription and hence its potential in regulating translation, we measured the level of protein encoded by the mRNAs that we examined by RT-PCR. For each genotype, RNA from four corneal epithelia was pooled and assayed for three times.

**Figure 5:**
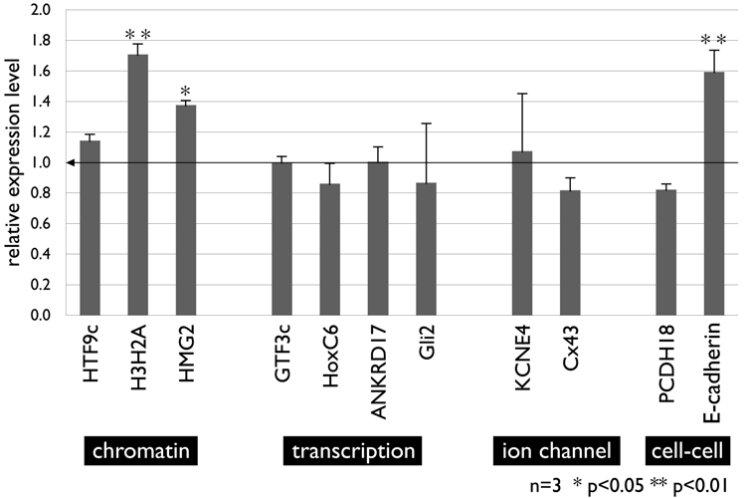
Transcript (mRNA) level of basonuclin-target genes in basonuclin-null corneal epithelium. Relative mRNA level of basonuclin-target genes was analyzed by real-time PCR using total RNAs isolated from the control and basonuclin-null corneal epithelia. Each RNA preparation was a mixture of four corneal epithelia of the same genotype and the analysis was performed in triplicates (n = 3). The average level of expression was normalized to beta-actin. The bar graph represents the average of three RNA preparations and their statistical variation (s.d.). The gene identity is given below each bar and the pathway of the genes indicated with white letters on a black background. * P<0.05, ** P<0.01.

We detected consistent and significant mRNA changes in two pathways, chromatin structure and cell adhesion. Two mRNAs in the chromatin structure pathways were up-regulated in basonuclin-null corneal epithelium; the mRNAs of histone 3 H2A (H3)(NM_178218) was up by ∼70% (n = 3, p<0.01) and that of the high-mobility group 2 (HMG2, NM_008252), ∼40% (n = 3, p<0.05). In cell adhesion pathways, the mRNA of E-cadherin (NM_009864) was up-regulated by ∼50%. Other perturbations in mRNAs were similarly detected in the pathways of transcription regulation and ion channel (**Fig. 5**), although these changes were not statistically significant.

**Figure 6:**
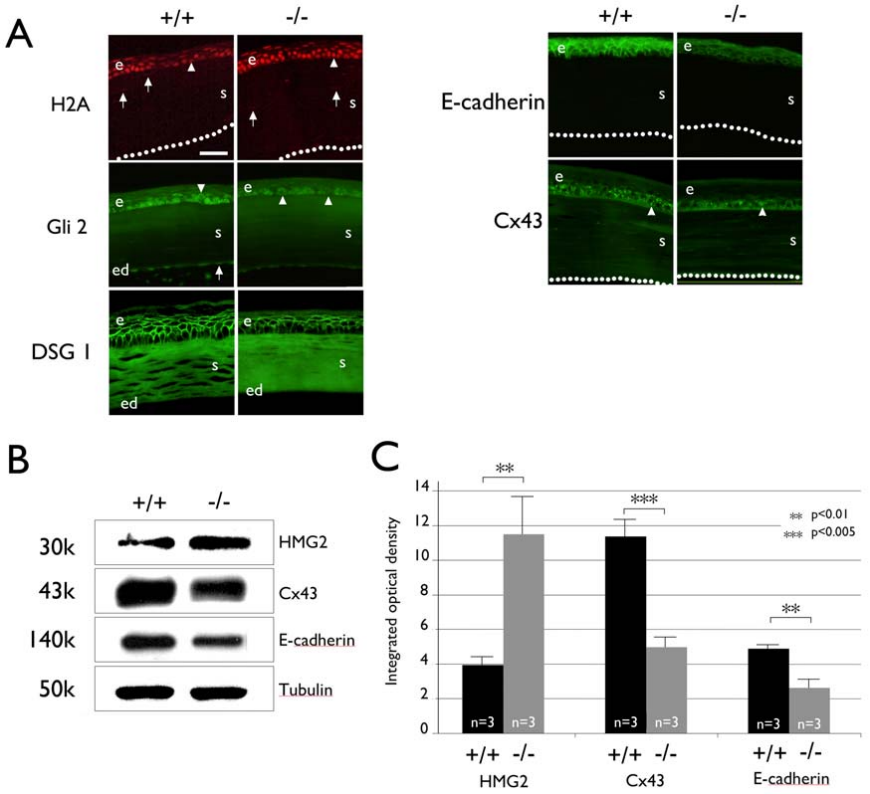
Basonuclin-null mutation affects level of proteins in basonuclin-targeted pathways. A, Immunostaining shows perturbation of protein level in basonuclin-null corneal epithelium. The antibody specificity is indicated on the left of immunofluorescent images and the genotype on the top. Arrowheads show examples of a positively stained cell in the epithelium and arrows that in the stroma and endothelium. Dotted-lines indicate the surface of the endothelium. e, epithelium, s, stroma, ed, endothelium. B, Immunoblot of selected proteins whose mRNA were perturbed by basonuclin-null mutation. The antibody specificity is shown on the right of the blot image and the genotype on the top. The apparent molecular weight of each detected protein is given to the left. C, Quantification of the Immunoblot analyses. The values are average of three measurements (n = 3). **, p<0.01; ***, p<0.005.

Assessing perturbation in proteins by immunocytochemistry and immunoblotting confirmed the changes detected in most of the mRNA, including those that were not statistically significant. Although we aimed to produce immunocytochemical and immunoblotting data for each protein, some antibodies failed to work in one procedure. Immunocytochemistry detected a stronger presence of histone 3 H2A in the basonuclin-null-corneal epithelium than in the keratocytes of the stroma (**Fig. 6A**). Similarly, immunoblotting showed an increase in HMG2 in basonuclin-null corneal epithelium (**Fig. 6B**). Both agreed with the RT-PCR results. Immunocytochemistry also showed a consistent down-regulation of Gli 2 protein, an effector of the Sonic Hedgehog (Shh) pathway. The decreased immunostaining agreed with the reduction of Gli2 mRNA detected by RT-PCR, albeit the mRNA change was not statistically significant, suggesting a specific but marginal decrease of Gli2 function in the basonuclin-null corneal epithelium.

Another group of basonuclin target genes was cell surface proteins. By RT-PCR, the transcript of Connexin 43 (Cx43), a gap-junction protein, was decreased nearly 20% in basonuclin-null corneal epithelium. This decrease was confirmed by immunoblot, which showed a 60% decrease of Cx43 protein (**Fig. 6A, B**). Immunostaining study showed a punctuated pattern of Cx43 in the basal cell layer of corneal epithelium. Scoring the number of Cx43 foci showed that the average number of foci in the control was 253±40, whereas that in the basonuclin-null mouse was 212±59 (n = 7, P<0.01), a 16% decrease in the mutant. Therefore, Cx43 function was impaired by basonuclin-null mutation. Our data showed previously that the promoter of desmoglein 1 (DSG1) gene, a component of desmosome, contained a basonuclin binding site [27] and DSG1 mRNA was upregulated 4-fold in basonuclin-deficient oocytes [26]. However, in basonuclin-null corneal epithelium, the levels of DSG1 mRNA and protein were not significantly altered (**Fig. 6A**), suggesting tissue/cell-type variation in response to basonuclin-deficiency.

The most interesting alteration was observed in adherens. We showed previously that one of basonuclin target genes was in the adheren family, i.e., proto-cadherin 18 (PCDH18), and the expressions of members of this family were perturbed in basonuclin-deficient oocytes [26], [27]. In corneal epithelium, quantitative PCR showed that basonuclin-null mutation caused an 18% decrease in PCDH18 mRNA level, and a 50% increase in the amount of E-cadherin mRNA (n = 3, P<0.01) (**Fig. 5**), but the distribution of E-cadherin, visualized by immunofluorescence, was not significantly changed (**Fig. 6A**). Interestingly, the immunostaining intensity suggested a reduction of E-cadherin protein level, contrary to the increase in its mRNA level (**Fig. 5** **and** **6B**). The contradictory changes of E-cadherin mRNA (up-regulated) and protein (down-regulated) were further investigated by quantitative analysis. Immunoblotting showed a highly significant two-fold decrease in E-cadherin protein (n = 3, p<0.01) (**Fig. 6B**). The non-concerted change of E-cadherin mRNA and protein suggested that the mRNA up-regulation was a compensatory adjustment in response to the decrease in the quantity of protein. Because E-cadherin's critical role in signal transduction and its linking role in cell adhesion and proliferation [6], [8], [38]–[40], the impaired E-cadherin seen in basonuclin-null corneal epithelium could at least in part explain the reduced cell proliferation in homeostasis as well as in wound repair.

## Discussion

Using a basonuclin-null mouse, we demonstrate for the first time the requirement of basonuclin in keratinocyte proliferation. Previously, based on basonuclin's basal location in the stratified epithelium and its presence in every stage of the cell cycle, including G0, we proposed that basonuclin played a role in keratinocyte proliferation [18]–[20]. Our study presented here confirms basonuclin's role in keratinocyte proliferation; it also suggests that basonuclin plays a role in the regulatory mechanism of cellular proliferation, but not the cell division process itself. This conclusion is deduced from the observation that basonuclin-null mutation does not affect neonatal corneal keratinocyte proliferation. During the formation of corneal epithelium in neonatal mouse (P1 to P14), corneal keratinocytes proliferate at a very high rate, and basonuclin expression is seen at the beginning of this proliferation period (i.e., around P3–P4) [21]. The coincidence of basonuclin's appearance and the rapid keratinocyte proliferation led us to postulate that basonuclin played a role in the high rate cell proliferation during corneal epithelium development. We were baffled, however, by the persistent high level basonuclin expression through out the adulthood after developmentally related proliferation had subsided post-eye-opening. Our study of basonuclin-null corneal epithelium provides a satisfactory resolution to this dilemma; basonuclin is required for the homeostasis but not development of corneal epithelium. In the basonuclin-null mouse, the neonatal development of this epithelium is not retarded, suggesting that basonuclin is not critically required in this process. Furthermore, this observation suggests that the defect of basonuclin-null mutation is not in the cell division mechanism. Cell proliferation is down-regulated after eye-opening around P14 in mouse, and is maintained at a low level during the rest of the life span [15], [21]. Our study suggests that basonuclin is required for maintaining an optimal level of cell proliferation necessary for corneal epithelium homeostasis, because we observed both a reduction in cell number and the proliferative (Ki67-positive) cells in the basonuclin-null corneal epithelium. An additional defect of basonuclin-null keratinocytes was revealed in the wound-healing process. In this case, the timing of the on-set of cellular proliferation is delayed, which suggest a signaling defect in mobilizing keratinocytes to respond the wound. The notion of a signaling defect is consistent with our hypothesis that basonuclin is a coordinator of multiple pathways. The slower mobilization of cellular proliferation therefore is likely due to the lack of coordination of various pathways participating in the mobilization process. Another logical conclusion is that the signaling (or coordination) mechanism controlling cell proliferation in neonatal corneal epithelium development differs from that functioning in adulthood for the homeostasis of this tissue.

The clear corneal phenotype of basonuclin (Bnc1)-null mutation suggests that it and basonuclin-2 (Bnc2), the second member of the basonuclin family, have non-overlapping functions. Bnc2, a closely related homolog of Bnc1, has a much wider tissue distribution than that of Bnc1 [41], [42]; and both are reportedly co-present in keratinocytes and oocytes [26], [41]–[43]. In Bnc1-deficient oocytes, in which Bnc2 expression is not affected, we also observed a clear phenotype (i.e., maternal effect), suggesting in mouse oocytes Bnc1 and 2 also have non-overlapping functions [26]. This notion is supported by a recent observation that Bnc1 and 2 reside in different intracellular compartments [44]. These observations, however, do not exclude the possibility that the two proteins share similar functions, because of the high homology of their zinc fingers and DNA targets [41]. Our current model systems (Bnc1-null and Bnc1-deficiency) are not suitable for addressing the issue of overlapping functions of the two proteins. The answer to this question awaits the development of a Bnc2-null mutation and the generation of a Bnc1-Bnc2 double null mutant.

**Figure 7:**
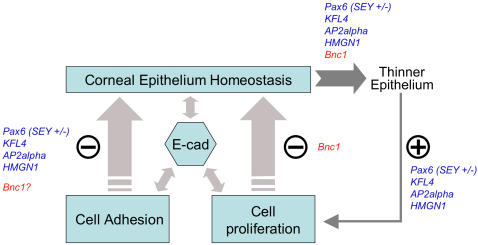
A model of maintenance of corneal epithelial homeostasis. The model asserts that cell proliferation and cell adhesion are major processes and E-cadherin a molecular regulator in maintaining corneal epithelium homeostasis. The model also proposes a regulatory feedback loop in controlling corneal epithelial thickness. Any perturbation (e.g., thinning) in corneal thickness would be counterbalanced by adjustment in the rate of cell proliferation. The model is based on previous reports on null mutations of Pax6, KFL4, AP2alpha, HMGN1 and this work (Bnc1) (See text for the description).

Several previous reports and our study suggest cell proliferation is a major process in maintaining corneal thickness. The thinner corneal epithelium in basonuclin-null mouse is similar to the phenotype of several recently described mouse models (i.e., null mutants of Pax6, KLF4, AP2-alpha and HMGN1). The thinning of corneal epithelium suggests that homeostasis is perturbed by these null mutations. However, the causes of this perturbation appear quite different. For example, in adult SEY(+/−), a heterozygotes of Pax6-null [8], the thinning of cornea epithelium is likely due to a compromised cell adhesion, which results in large gaps between epithelial cells and a change in desmosomal junctions [8]. In another example, the conditional null mutation of Krüppel-like transcription factor-4 (KLF4) reduces the corneal epithelium to only three to four cell layers by causing excessive sloughing [45]. The third example concerns the conditional deletion of AP-2alpha (Le-AP-2alpha), which leads to a variable thickness of corneal epithelium (2 to 10 cell layers), and a thinner and discontinuous basement membrane [39]. The null mutation of HMGN1, a member of the high-mobility group (HMG) proteins, gives rise to corneal epithelium thinning, which appears to be the consequence of an impaired keratinocyte differentiation program [38]. Notably, in the aforementioned four null mutants (i.e., SEY (+/−), KLF4-null, Le-AP-2alpha-null and HMGN1), an up-regulation of proliferation in corneal epithelia is invariably observed, suggesting the existence of a feedback loop that regulates corneal thickness [8], [39]. When corneal epithelium becomes thinner due to deficiency in cell adhesion or other causes, cell proliferation is increased to compensate. Our analysis of basonuclin-null corneal epithelium provides the first evidence from another angle (i.e., down-regulation of cell proliferation) to support this model. Based on this analysis, we propose that under normal homeostasis, cell proliferation is constantly adjusted to counterbalance occasional perturbation in corneal thickness (**Fig. 7**). This proposal implies a monitoring mechanism of corneal homeostasis and a feedback mechanism to tune cell proliferation rate according to corneal thickness. A regulatory role of basonuclin in fine-tuning cell proliferation is consistent with basonuclin's location in basal keratinocytes of stratified epithelium.

Although the molecular nature of such mechanisms remains to be elucidated, the aforementioned studies suggest a central role of E-cadherin in maintaining the homeostasis of corneal epithelium. The histological and cell biological manifestations of the aforementioned null-mutations are different, some affecting cell adhesion, and others cell differentiation or cell proliferation, suggesting they impair distinctive aspects of corneal epithelium homeostasis. Remarkably, they all perturb the expression of E-cadherin [8], [38], [39], [45](this work), suggesting this protein is at the pivotal point of regulation of corneal epithelial homeostasis. E-cadherin is indeed capable of influencing both cell adhesion and nuclear events and other pathways through its interaction with beta-catenin and EphA2 receptor tyrosine kinase [46]–[49]. E-cadherin/beta-catenin complex is also regulated by tyrosine phosphorylation [49] and is a target of Wnt signaling pathway [50]. The convergence of a wide range of interactions on E-cadherin is consistent with a role of a sensor and information integrator in intercellular and intracellular events (**Fig. 7**). A perplexing observation is that in most cases, a down-regulation of E-cadherin function leads to an increase in proliferation, but we found that basonuclin-null mutation resulted in a down-regulation of E-cadherin and a decrease in proliferation.

Although there is a strong correlation of E-cadherin function perturbation and the thinning of corneal epithelium, the underlying molecular alterations of these null-mutations (i.e., Pax6, KLF4, AP-2alpha, HMGN1 and Bnc1), which lead to changes in E-cadherin function, are likely different. In a sense, basonuclin is unique because none of the other aforementioned null-mutations has been shown to regulate rDNA transcription. In our examination of the v-rDNA expression profile in corneal epithelium, we made two novel findings. First, we established the v-rDNA expression profile in corneal keratinocytes. We found that v-rDNA VII, whose transcript was absent in most of the tissues (Tseng et al., submitted), was expressed in cornea keratinocytes. Second, we identified the v-rDNA perturbed by basonuclin-null mutation (v-rDNA I, II and VI). These findings support the notion that cell-type-specific regulation of rDNA relates to the differential usage of a subset of rDNA [25], [51]. However, currently it is not understood why v-rDNAs are expressed differentially in various tissues, or whether these v-rDNAs encode different ribosomal RNA (rRNA). The lack of knowledge in this area precludes an understanding of why basonuclin can regulate both Pol I and Pol II transcription and why it regulates a subset of rDNA. Nevertheless, our data strongly suggest that basonuclin-null mutation impairs the expression of E-cadherin. Our results show that the amount of E-cadherin protein was reduced in basonuclin-null corneal epithelium but its mRNA level was increased. It can be argued that the decrease in the amount of E-cadherin protein is not due to an impairment of transcription and basonuclin-null keratinocytes are capable of increasing production of E-cadherin mRNA, probably in an attempt to compensate for the reduced protein level. The defect thus appears to be post-transcriptional and likely translational, because of basonuclin's role in rRNA synthesis and ribosomal biogenesis. The implication of this interpretation is that basonuclin-null mutation affects E-cadherin translation selectively, because we did not detect contradictory changes in mRNA and protein levels in other genes (e.g., Gli2, HMG2 and Cx43). Therefore, the mechanism, by which basonuclin-null mutation perturbs E-cadherin expression, warrants future investigation.

In summary, we describe the construction of the first basonuclin-null mouse model and the characterization of its corneal epithelium. Basonuclin-null mutation impairs regulation of corneal keratinocyte proliferation, which leads to perturbation in epithelium thickness and delay in wound re-epithelialization. Our data is consistent with the notion that basonuclin regulates pathways requiring both Pol I and II transcription. Importantly, our result confirms that basonuclin regulates a subset of rDNA, and suggests the identity of basonuclin's target v-rDNA. Based on reports by others and this study, we propose a regulatory feedback loop in maintaining corneal epithelium thickness, and that basonuclin and E-cadherin are important regulators in corneal epithelium homeostasis (thickness).

## Materials and Methods

### Generation of basonuclin-null mutant mice

The exon' 4 of the basonuclin gene, which encodes the critical zinc fingers and the nuclear localization signal, was targeted for excision by the Cre recombinase. The targeting vector, which was constructed by ET recombination [52] with a 12.7kb genomic sequence, was introduced into 129Sv/R1 ES cells [34] by electroporation. Homologous recombination was detected by Southern hybridization. Two independently isolated Bnc1 (fl-neo/+) clones were injected into C57BL/6J blastocysts at the Transgenic & Chimeric Mouse Facility at PENN. One clone was germ-line transmitted. Chimeric males were crossed with C57BL/6J females to generate Bnc1 (fl-neo/+) mice. Bnc1 (+/−) mice were obtained by crossing a normal male with a Bnc1 (fl-neo/+: ZP3-Cre) female according to the scheme described in Fig. 2a of [35]. Southern hybridization and PCR analysis confirmed the genotype (for PCR primers, see Table S1). Bnc1 (+/−) mice were maintained on a 129Sv/Sv-CP/C57BL/6J hybrid genetic background, and used for generating Bnc1 (−/−).

### Tissue Preparations

All animal procedures were performed in accordance with the ARVO Statement for the Use of Animals in Ophthalmic and Vision Research. The littermates (Bnc1 +/+) of basonuclin-null mice were used as control. For RNA or protein extraction, eye globes of 12-week old mice were dissected and the corneas were soaked in 20 mM EDTA at 37°C for 1 hr. The epithelium was gently pealed off and frozen immediately in liquid nitrogen. For histology, immunocytochemical staining or apoptosis assay, whole eye globes were surgically removed from 12-week old mice after euthanization and immediately embedded in O.C.T. (Tissue-Tek®, Sakura Finetec USA, Inc, Torrance, CA) and frozen or fixed in alcoholic formalin [53] at 4°C overnight before being processed for serial dehydration and paraffin embedding.

### RNA Isolation and PCR

Total RNA was isolated from a mixture of four corneal epithelium sheets using TRIzol Reagent (Invitrogen, Carlsbad, CA) according to the manufacturer's instructions. For v-rDNA expression study, 2 µg total RNA was reverse transcribed with random primers using the SuperScript First-Strand Synthesis System of Invitrogen. Each variant-specific PCR was performed with a unique pair of primers, which conveyed the specificity. For each v-rDNA, PCRs were always performed together as a set, which was comprised of specificity controls using cloned v-rDNA as templates, and the sample RNAs. This scheme ensures that each sample RNA is assayed with the required specificity and no cross-detection of v-rDNA occurs. The cycling conditions were also optimized so that all variant-specific PCRs ran with similar efficiency. The primer sequences and PCR conditions will be provided upon request.

For mRNA transcription assay, specific primers were designed for each target gene using Primer3 software (http://primer3.sourceforge.net/) and the cDNA sequence in GenBank (Table S2). Total RNA (1 µg) was reverse-transcribed with oligo-dT primers using the SuperScript First-Strand Synthesis System. The resulting single-stranded cDNAs were quantified by real-time PCR on a DNA Engine OPTICON2 (MJ Research, Cambridge, MA) with DyNAmo SYBR Green qPCR Kit (F-400S, New England BioLabs, Ipswich, MA). Beta-actin mRNA was used as internal reference. Thermocycling parameters were 95°C 10 minutes, then 40 cycles of 94°C for 30 seconds, 60°C for 40 seconds, 72°C for 40 seconds. Each sample was analyzed in triplicate, the average of which was normalized against the expression level of beta-actin before statistical analysis.

### Immunoblotting

Total protein was extracted from two corneal epithelium sheets of each mouse and lysed in RIPA buffer (i.e., 1% NP-40, 0.5% sodium deoxycholate, 0.1% SDS, 5mM EDTA) with 100 ng/ml protease inhibitor cocktail (Roche, Indianapolis, IN). Protein concentration was determined using the Bio-Rad Protein Assay (Bio-Rad Laboratories, Hercules, CA). Proteins were separated on a SDS-PAGE (4∼15% gradient Tris-HCL, Bio-Rad Laboratories) and transfered to a PVDF membrane using a semi-dry electrophoretic transfer device (Bio-Rad Laboratories) and the 3-buffer system (Millipore, Billerica, MA). The membrane was blocked with 5% non-fat dry milk (Bio-Rad Laboratories) in Tris-buffered saline at room temperature for 1 hour and incubated with primary antibody at 4°C overnight. The membrane was washed and incubated with one of the secondary antibodies, depending on the origin of the primary antibody. The secondary antibodies were anti-rabbit, anti-mouse, or anti-rat horseradish peroxidase-conjugated (1∶5000, GE Healthcare, Piscataway, NJ). The immunocomplex was visualized using an ECL kit (Amersham/GE Healthcare). The following primary antibodies were used, anti-Cx43 (1∶2000, Sigma-Aldrich Corp), anti-E-cadherin (1∶500, Invitrogen), anti-HMG2 (1∶1000, Calbiochem, San Diego, CA) and anti-beta-tubulin (1∶10000, Sigma-Aldrich Corp).

### Histology and DAPI Staining

Five-micron paraffin sections were stained with hematoxylin and eosin (H&E) for histological analysis. Slides were counterstained with DAPI and photographed using a Nikon Eclipse TE2000-U microscope and a Nikon D1 camera. The number of DAPI-stained nuclei of each cornea was determined at the central cornea (400× field, or approximate 0.3 mm). Six mice of each genotype were analyzed.

### Immunofluorescence staining

Six-micron cryosections were fixed for 5 minutes each in methanol (−20°C) and acetone (−20°C). Sections were permeabilized by 0.01% Triton X-100 before incubation with primary antibody at 4°C overnight. The following primary antibodies were used: anti-basonuclin (1:30) [25], anti-E-cadherin (1∶100). Five-micron paraffin sections were de-paraffinized, re-hydrated and then washed in PBS. After antigen un-masking treatment, sections were blocked with 5% normal goat serum and incubated with primary antibody at 4°C overnight. The antibodies used are as follows: anti-Cx43 (1∶500), anti-DSG1 (1∶400) [54], anti-Gli2 (1∶50, Abcam, Cambridge, MA), anti-Ki67 (1∶50, Vector Laboratories, Burlingame, CA). The next day, slides were washed in PBS containing 0.1% NP-40 and incubated with a secondary antibody (anti-rabbit-Alexa Fluor 488, anti-rat-Alexa Fluor 488, anti-mouse-Alexa Fluor 594, Invitrogen) at room temperature for 1 hour. All sections were mounted in anti-fade mounting medium with DAPI (Vector Laboratory) to reveal nuclear staining. For each experiment, a section was stained without primary antibody to serve as a negative control. All staining was documented as described in Histology and DAPI staining. Particle analysis function of the Image J software (version 1.36b, NIH) was used to analyze Cx43 foci. Sections from six mice of each genotype were analyzed.

### Apoptosis Assay

TUNEL (TdT-mediated dUTP nick end labeling) assay on paraffin sections was carried out with the In Situ Cell Death Detection Kit, Fluorescein (Roche) according to the manufacturer's instruction. Slides were mounted in anti-fade medium with DAPI and examined with the Nikon fluorescence microscope.

### Corneal Epithelium Wounding and Re-epithelialization

Control and basonuclin-null mice (11 to 12 weeks old) were anesthetized by an intraperitoneal injection of 80 mg/Kg ketamine hydrochloride and 16 mg/Kg xylazine. Residual eye reflexes were blocked by topical application of 0.5% proparacaine hycrochloride (Alcon Laboratories, Fort Worth, TX) to the corneal surface. A punch (Acuderm Inc., Ft. Lauderdale, FL) was used to mark a 2 mm diameter circular area at the center of the cornea under a dissecting microscope. The corneal epithelium was debrided with an Algerbrush corneal rust ring remover with a 0.5 mm burr (Katena Products Inc., Denville, NJ). Antibiotic ointment was applied to the eyes after surgery. The epithelial lesions of both the control and basonuclin-null eyes were topically stained with fluoress (Akorn, Buffalo Grove, IL) and photographed at various time points post-wounding to monitor re-epithelialization. The circumference of the wound margin was traced and the wound area was calculated. The data are presented as (A0-A)/A0)*100, in which, A is the wound area at a given post-wounding time point and A0, the wound area at time 0. Some wounded eyes were removed after BrdU injection to examine the onset of DNA replication (see below).

### DNA Replication Assay

Mice were injected intraperitoneally with BrdU at a dose of 20 µg/g body weight and euthanized by CO_2_ inhalation 120 minutes post-injection. Eye globes were dissected and processed for 5 µm paraffin sections. The sections were treated with proteinase K for 10 minutes at 37°C followed by incubation in 2N HCl for 50 minutes at 37°C. The slides were blocked in 5% BSA, and incubated with a rat anti-BrdU antibody (AbD Serotec, Raleigh, NC) at 4°C overnight. The BrdU labeling was visualized by an anti-rat-IgG-Alexo Fluro488 antibody (Invitrogen). The number of BrdU-positive cells was scored in three corneas for each genotype (n = 3).

### Statistical Analysis

Two-tailed Student's t-tests (Excel, Microsoft, Redmond, WA) was used in analyzing the quantitative PCR data, the cell number scoring, the number of Ki67-positive cells. Chi-square tests were employed to analyze the foci of Cx43. Null hypotheses were rejected whey p<0.05. All quantification data are presented as mean±sd.

## Supporting Information

Table S1 PCR primers
(0.03 MB DOC)

Table S2 PCR primers
(0.03 MB DOC)
