# Supplementary material for: Basonuclin-Null Mutation Impairs Homeostasis and Wound Repair in Mouse Corneal Epithelium
Source: PLoS One. 2007 Oct 31;2(10):e1087. doi: 10.1371/journal.pone.0001087 (PMC2034529; doi:10.1371/journal.pone.0001087)
Supplement: Table S1 — PCR primers (0.03 MB DOC) [file pone.0001087.s001.doc]

Table S1. Sequences of primers for Bnc1

| Primers | Sequence |
| --- | --- |
| P1 | gct ctt cta ggc cag tgg tg |
| P2 | ccc caa acc agc agt ttt ta |
| P3 | cca tcg cta acc tga gaa cc |
| Pair D (forward) | tcg ctt tgg aga gac caa gt |
| Pair D (reverse) | ctc ctc agg gag ctg aac ac |
| Pair A (forward) | gcc gtc cac cta aag atc aa |
| Pair A (reverse) | gtc ctc tgg ctc gtc ttc ac |
