# Supplementary material for: Basonuclin-Null Mutation Impairs Homeostasis and Wound Repair in Mouse Corneal Epithelium
Source: PLoS One. 2007 Oct 31;2(10):e1087. doi: 10.1371/journal.pone.0001087 (PMC2034529; doi:10.1371/journal.pone.0001087)
Supplement: Table S2 — PCR primers (0.03 MB DOC) [file pone.0001087.s002.doc]

Table S2. Sequences of primers for quantitative PCR

| Genes | NCBI locus number | Forward primer | Reverse primer |
| --- | --- | --- | --- |
| Htf9c | NM_008307 | cat ctc ccc tca tgc att ct | ggc aca gtt caa ttc cca ct |
| Hist3H2a | NM_178218 | gca aaa gcg aag tct cgt tc | ctt gtt gag ctc ctc gtc gt |
| Hmg B2 | NM_008252 | atg ctc cga gag atg gaa ga | ttg atc ttt ggg cga ttt tc |
| GTF3c1 | NM_207239 | tct ccg cct tgg aga aga ta | agc atc agt ctc agg gca gt |
| HoxC6 | NM_010465 | tcc aga ttt acc cct gga tg | tgc tct tct cgc ttt tcc tc |
| Ankrd17 | NM_030886 | ggg aag aag gat gga agg ag | gga att tgc tgt gga gct tt |
| Gli2 | XM_973171 | cgc act cac tcc aat gag aa | gga cat gca cat cat tac gc |
| Pcdh18 | NM_130448 | tag aca ggc tcc tgg gag aa | tga gaa tgc tgt tgc tga gg |
| E-cadherin | NM_009864 | act gtg aag gga cgg tca ac | gga gca gca gga tca gaa tc |
| Cx43 | NM_010288 | gtg gcc tgc tga gaa cct ac | gag cga gag aca cca agg ac |
| Kcne4 | NM_021342 | aag agg cgg gag aag aag tc | ctc aca ctg tcc cct tcc at |
